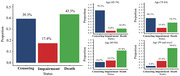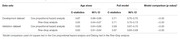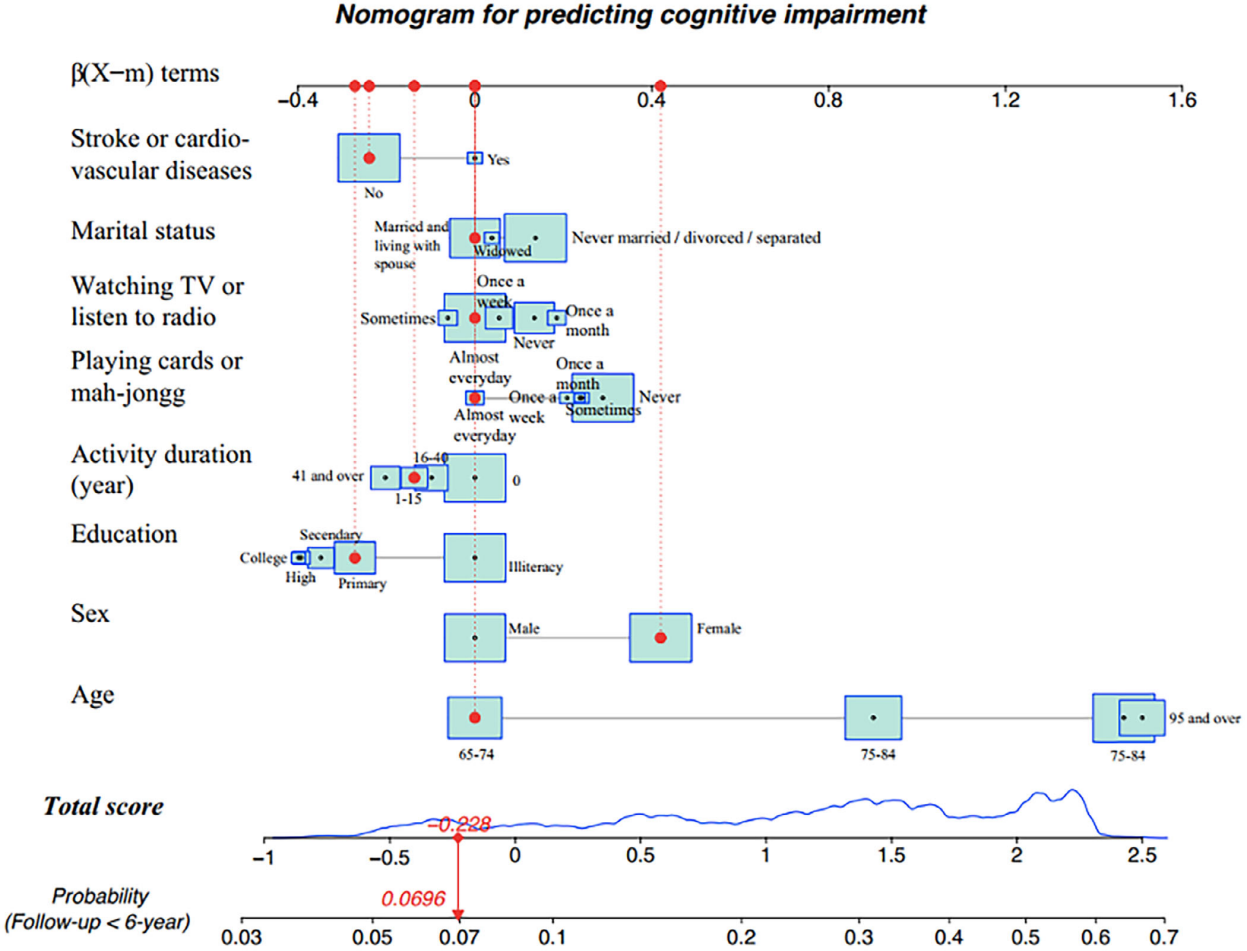# Derivation and Validation of the Cognitive Impairment Prediction Model in Older Adults: A National Cohort Study

**DOI:** 10.1002/alz.084173

**Published:** 2025-01-09

**Authors:** Mingyue Hu

**Affiliations:** ^1^ Central South University, Changsha, Hunan China

## Abstract

**Background:**

This prediction model quantifies the risk of cognitive impairment. This aim of this study was to develop and validate a prediction model to calculate the 6‐year risk of cognitive impairment.

**Methods:**

Participants from the Chinese Longitudinal Healthy Longevity Survey (CLHLS) 2008‐2014 and 2011‐2018 surveys were included for developing the cognitive impairment prediction model. The least absolute shrinkage and selection operator, clinical knowledge, and previous experience were performed to select predictors. The Cox proportional hazard model and Fine‐Gray analysis adjusting for death were conducted to construct the model. The discriminative ability was measured using C‐statistics. The model was evaluated externally using the temporal validation method via the CLHLS 2002‐2008 survey. A nomogram was conducted to enhance the practical use. The population attributable fraction was calculated.

**Results:**

A total of 10,053 older adults were included for model development. During a median of 5.68 years, 1,750 (17.4%) participants experienced cognitive impairment. Eight easy‐to‐obtain predictors were used to develop the model. The overall proportion of death was 43.3%. The effect of age on cognitive impairment reduced after adjusting the competing risk of death. The Cox and Fine–Gray models showed a similar discriminative ability, with average C‐statistics of 0.71 and 0.69 in development and external validation datasets, respectively. The model performed better in younger older adults (65‐74 years). The proportion of 6‐year cognitive impairment due to modifiable risk factors was 47.7%.

**Conclusion:**

This model could be used to identify older adults aged 65 years and above at high risk of cognitive impairment and initiate timely interventions on modifiable factors to prevent nearly half of dementia.